# Kinetics of Lipophilic Pesticide Uptake by Living Maize

**DOI:** 10.1021/acsagscitech.3c00042

**Published:** 2023-04-18

**Authors:** Joseph R. Elliott, Joseph Cortvriend, Giovambattista Depietra, Colin Brennan, Richard G. Compton

**Affiliations:** † Department of Chemistry, Physical and Theoretical Chemistry Laboratory, University of Oxford, South Parks Road, Oxford OX1 3QZ, Great Britain; ‡ Jealott’s Hill International Research Centre, Syngenta Ltd., Bracknell, Berkshire RG42 6EY, Great Britain

**Keywords:** foliar uptake, cuticle, diffusion, simulation, formulation

## Abstract

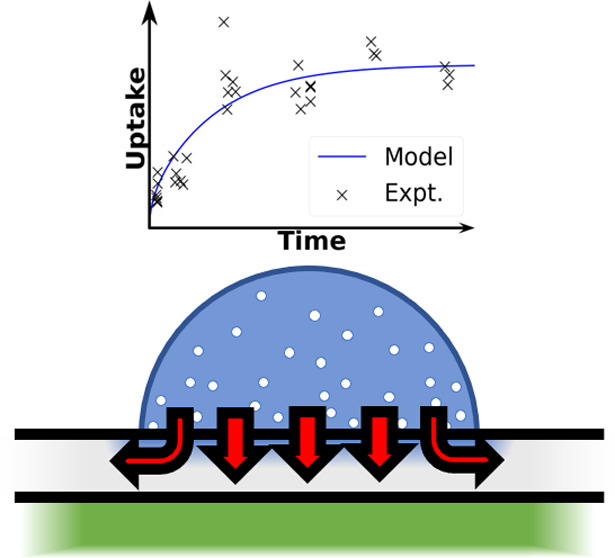

We report the uptake of a lipophilic fungicide into the cuticle of living leaves of young maize from droplets of a suspension concentrate. The action of a “coffee-ring” effect is demonstrated during fungicide formulation drying, and the fungicide particle distribution is quantified. We develop a simple, two-dimensional model of uptake leading to a “reservoir” of cuticular fungicide. This model allows inferences of physicochemical properties for fungicides inside the cuticular medium. The diffusion coefficient closely agrees with literature penetration experiments (*D*_cut_ ≈ 10^–18^ m^2^ s^–1^). The logarithm of the inferred cuticle–water partition coefficient log_10_ *K*_cw_ = 6.03 ± 0.04 is consistent with ethyl acetate as a model solvent for the maize cuticle. Two limiting kinetic uptake regimes are inferred from the model for short and long times, with the transition resulting from longitudinal saturation of the cuticle beneath the droplet. We discuss the strengths, limitations, and generalizability of our model within the “cuticle reservoir” approximation.

## Introduction

Understanding the uptake of lipophilic pesticides into the cuticles of agronomically significant plant species is of vital importance from the perspectives of agrochemical efficiency,^1−3^ environmentalism,^4−7^ and toxicology,^7−10^ including risk assessment and food quality. The lipidic cuticle is the plant’s primary barrier against the uptake of potentially hazardous material, including pesticides.^2,3,11,12^ Not only does it sequester lipophilic material^13^ and thus can reduce uptake into the underlying tissue, but it also acts as a diffusional barrier,^2^ slowing the mass transport. Agrochemicals, especially pesticides, are frequently applied as water-based foliar sprays^14^ and thus must interact with the cuticle prior to their function. Understanding the behavior of lipophilic material in and on the cuticle facilitates the design of these products for efficiency and safety. Generally, rapid and effective uptake into the cuticle is preferred, either as a precursor to transport into the plant tissue^2^ (e.g., systemic herbicides) or to enhance the pest resistance of the cuticle itself (e.g., certain fungicides and insecticides). Rapid uptake from the leaf surface may also help to mitigate loss processes from the leaf surface, such as washing off by rain, which also adds to the environmentally damaging effects of pesticide runoff.^7,15^ Additionally, cuticular uptake is vital for modeling the movement and accumulation of pesticides and other pollutants in the biosphere^8−10,13,16−19^ and thus assessing their threat to humans^8−10^ or to the environment,^13^ such as through crop consumption.

Developing effective models for the interaction of pesticide residues with plant cuticles^20−25^ provides insight into how future formulations can be tuned to demonstrate desired behavior,^1,26^ such as reduced risk profiles and greater food quality and yield, as well as identifying and explaining the observed behaviors of formulations used today. In particular, further work is required for modeling the behavior of particulate- and suspension-based formulations on cuticle surfaces, as these represent significant potential for delivery control, ease of use, and safety for the user and environment.^14,27−29^ This paper builds upon our prior publication^30^ working toward the effective modeling and simulation of these systems, using the previously developed theory to interpret experimental data.

Specifically, in this paper, we present experimental results for the uptake of an agronomically relevant, lipophilic fungicide into the cuticle compartment of living maize when applied as droplets of a simple, non-adjuvant suspension concentrate (SC) formulation. We present data extracted from microscopy to describe the deposits after dry-down, and using information inferred here and in the literature, we present a model for the uptake behavior. We discuss what can be inferred from this case study and discuss the generalizability of this model for the uptake of lipophilic particulate residues within a “cuticle reservoir” approximation.

## Experimental Method

### Uptake Experiments

Experiments to kinetically monitor the uptake of lipophilic fungicide were made by applying aqueous microdroplets containing known concentrations of fungicide to the leaves of two varieties of living maize plants.

Stock fungicidal formulation was acquired from Syngenta Ltd as a non-adjuvant suspension concentrate (SC). The formulation was chosen to minimize additive effects that might influence the uptake, such as adjuvancy.

Coxximo and Farmfire maize (Syngenta) were grown under ideal greenhouse conditions of 24 °C (day) and 18 °C (night) with ∼15 daylight hours and 55% humidity. 24 h before application of fungicide, they were transferred to a Weiss Gallenkamp Fitotron CE growth room, where prior conditions were maintained but at 70% humidity. 600 lumen light substituted daylight. Plant positions were randomized after transfer and before droplet application to mitigate any inhomogeneity in the growth conditions.

20 droplets of 0.2 μL were applied in a regular array to the leaf surface as approximate hemispheres of ∼460 μm radius. The droplets were applied to the adaxial surface halfway along the 2nd oldest leaf at 9 days old. The SC formulation was diluted to 375 ppm using 90: 10 v/v water: isopropyl alcohol. Droplets were applied with ≥3 mm separation to ensure diffusional independence in the leaf, as discussed below. A Sartorius Picus Biohit Single-Channel Electronic pipette was used.

To monitor the transfer of fungicide from the leaf surface into the leaf cuticle, the fungicide within each of these two compartments—surface and cuticle—was extracted after an exposure time. Each exposure time point refers to the time between application and extraction. 2 batches of 3 repeats were used for each time point, and the batch order was randomized. The time taken for the droplets to evaporate to dryness was also noted.

For extracting from the leaf surface, the whole leaf was washed for 20 s with 5 mL of 80:20 water/acetonitrile. For extracting from within the leaf, the leaf was dried, macerated with 5 mL of 20:80 water/acetonitrile (2 × 20 s at 6000 RPM using Precellys Lysing tube CK28 and Precellys Evolution homogenizer), and centrifuged (15 min at 4000 RPM using Heraeus Multifuge 3C). 1 mL of subsamples were taken. Quantitation of the fungicide in a subsample was performed by calibrated LC-MS using a Vanquish LC system feeding into ThermoFisher Q-Exactive UHMR.

Preliminary experiments involved washing with heptane after the surface wash to differentiate fungicide within the cuticle from fungicide within the subcuticular tissue, but this was observed to not provide statistically valuable quantitative information as shall be described in the Experimental Results section.

Statistical analysis involved ANOVA testing^31^ followed by post-hoc Tukey testing and calculation of covariance and Pearson and Spearman correlation coefficients; this analysis was performed in Python using the numpy, scipy, and statsmodels packages with the matplotlib package for plotting.

### RLM and cryo-SEM Surface-Deposit Characterization

Imaging was used to characterize the distribution of solid material remaining on the leaf surface following evaporation of the droplet arrays.

Two techniques were applied: reflected light microscopy (RLM) using Zeiss Axio Zoom. V16 and Zeiss ZEN software and cryogenic scanning electron microscopy (cryo-SEM) with a preparation procedure described below. All leaves for both techniques were imaged 1 h after droplet application.

To perform the cryo-SEM, ca. 1 cm × 1 cm sections were mounted on a Quorum specimen shuttle using Tissue-Tek O.C.T. compound. The sample was plunge-cooled in a liquid nitrogen slush before transfer to the Quorum PP3010T preparation chamber. The specimen holder was located onto the nitrogen gas-cooled sample stage maintained under vacuum at −120 °C. The sample was sublimated at −90 °C for 3 min prior to coating with gold/palladium for 60 s with a current of 5 mA and a temperature of −120 °C. The specimen shuttle was transferred to the Hitachi SU8220 Field Emission Scanning Electron Microscope (FESEM), and the sample was maintained at a temperature of −160 °C.

The open-source Fiji package^32^ for ImageJ2^33^ was used for image analysis. Semi-automated segmentation of cryo-SEM images using Trainable Weka Segmentation^34^ was trained by manual segmentation of a representative image into four classes: cuticle above palisade cell, cuticle above cuticular peg, damaged cuticle, and fungicide particle. The classifier consisted of a random forest of 200 trees using mean and bilateral filters.

## Experimental Results

In the following, we first present results for the characterization of the adaxial Coxximo maize leaf surface using the cryo-SEM technique described above. We then present results from observations of the evaporation times of the fungicidal droplets on this surface, followed by characterization of the resultant deposition of solid material by RLM and cryo-SEM, using the methods described above. These data are essential for the interpretation of the uptake kinetics and the development of suitable models, both of which are considered below.

### Leaf Characterization

**Figure 1:**
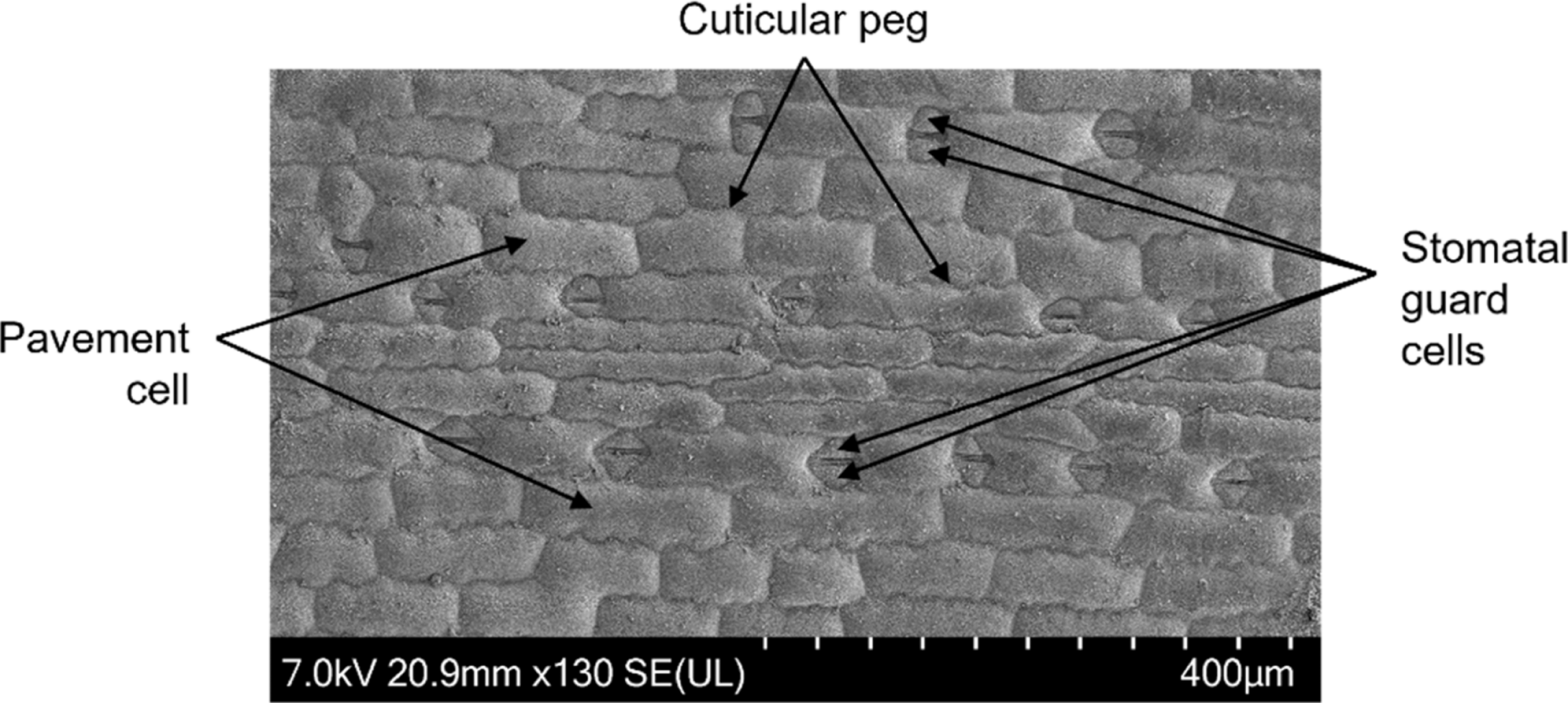
Cryo-SEM image of the interior of a dried deposit illustrating the cuticle above a leaf structure consisting of rows of pavement cells, stomatal complexes, and cuticular pegs. Image magnification was 130×. A scale bar of 400 μm is included at the bottom of the image.

Characterization of the leaf surface, performed by cryo-SEM and subsequent image analysis, was essential for informing the surface topology through which uptake occurs. The leaf surface of Coxximo maize was differentiated into the pavement and stomatal cells only, with cuticular pegs evident between these cells. No trichomes were observed. Epicuticular wax crystals were observed across the entire surface. Figure 1 demonstrates the leaf structure as observed by cryo-SEM.

Pavement cells (see Figure 1) provide protection for the more specialized cells beneath them and spacing between the stomata. We observe that the cuticle above pavement cells makes up the majority of the leaf’s surface area^35^ and is thus the predominant barrier to astomatous lipophilic uptake.^11,36,37^ The pavement cells were approximately rectangular with rounded vertices, major axes parallel to the leaf’s major vein, waviness in the shape outline, and a mean axis length ratio of ∼3. The cells were approximately organized into rows of alternating phases.

Stomatal cells, indicated in Figure 1, are specialized epidermal cells ubiquitous to plants that control the exchange of gas and water vapor between the environment and the leaf’s porous interior through the opening and closing of stomatal pores.^35^ They introduce surface inhomogeneity and potentially provide a stomatal uptake route for low-surface-tension mixtures.^38−40^ The stomatal complexes were observable by the two guard cells at the surface. All stomata observed by cryo-SEM were closed. The complexes were generally aligned with the pavement cell rows and shared the same width. The stomata were generally separated by one pavement cell only. The rows that contained stomata were randomly distributed but non-clustered.

The cuticular peg between all epidermal cells can be observed by its deformation of the cuticular topography into a slight trough. The width of the trough indicates the peg’s width at the leaf’s surface and may be used in approximating the cuticular peg volume. This deformation allows differentiation between the cells below the cuticle.^35^ The cuticular peg represents a region of greater cuticular depth and roughness of the internal cuticle–cell interface.^41^

The epicuticular waxes provide a barrier to surface wetting and reduce direct contact between solid particles and the cuticle. Epicuticular wax crystals were observed as plate-like fragments and needles with their major axes perpendicular to the underlying smooth wax surface. The exposed lengths of each were ∼1 μm, and their thicknesses were ∼0.05 μm.

Further quantitative details for this characterization are provided in the Supporting Information.

### Droplet Evaporation

Visual observation of the evaporation of the fungicidal droplets was performed to measure the window of time in which the formulation’s aqueous phase is present on the surface after application. The aqueous phase contributes to the uptake by offering an indirect pathway via dissolution and aqueous transport. The evaporation of the droplet induces convective flow, which mixes any dissolved material and directs the deposition of solid material.

**Figure 2:**
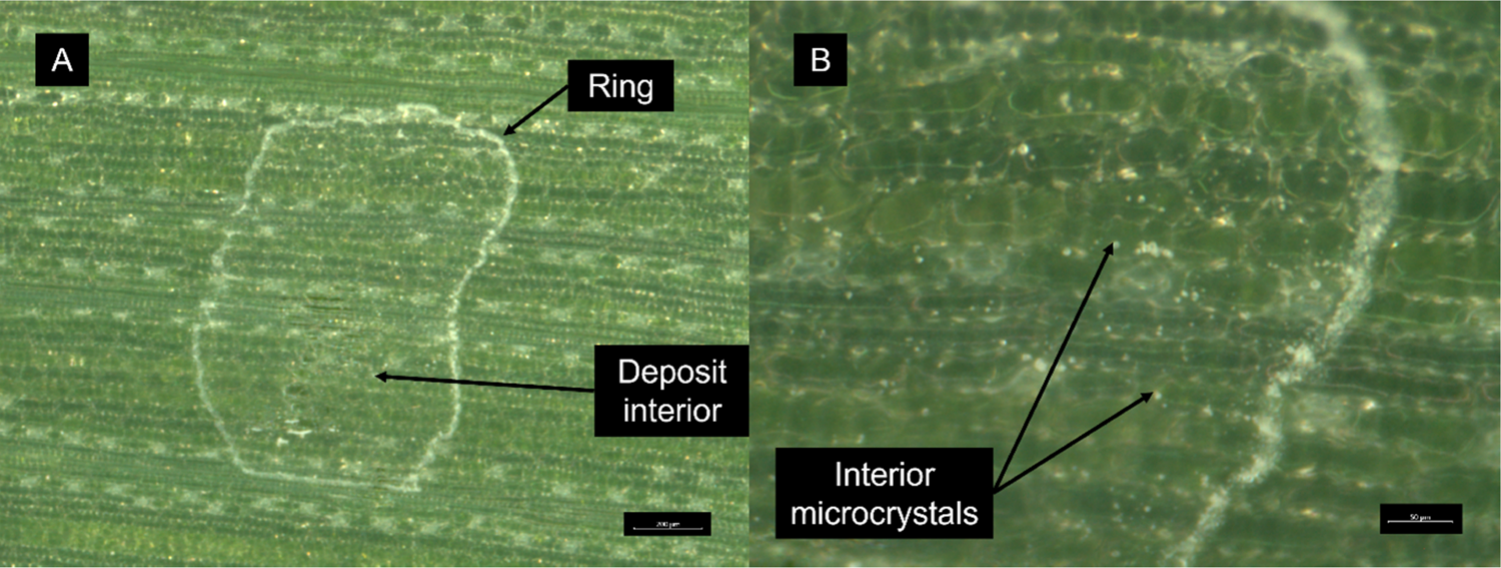
Annotated Reflected Light Microscopy images of dried deposits 1 h after application. Image A is at 40× magnification, and image B is at 150× magnification. Scale bars are in the lower right of each image (200 μm for A and 50 μm for B). Image B shows the presence of microcrystals within the ring’s interior, though at greater sparsity than within the ring itself.

The evaporation times of 5 arrays of 20 droplets of 0.2 μL containing fungicide on living Coxximo leaves, with ≥3 mm separation, were recorded. Droplet evaporation to dryness occurred 652 ± 3 s (10.9 ± 0.05 min) after application. For each droplet, a ring of deposited solid was visible to the eye after drying, with a predominance of material at the edge of the droplet’s contact area. Microcrystals of fungicide were randomly deposited within the interior area. Figure 2 demonstrates representative RLM images at 40× and 150× magnifications. The ring shape of the deposit is visible as is the presence of microcrystals within the interior.

### Deposit Characterization

The deposition of solid material after droplet drying indicated incomplete fungicide uptake prior to drying, and the deposit’s characterization by microscopy informs the uptake modeling, the physical processes that occur during evaporation, and the fungicide’s behavior after drying.

The deposit was characterized using RLM, where the deposit area was identified by its contrasting whiteness and high reflectivity, and cryo-SEM imaging, where the ring was seen as a greater concentration of particles and additive film with a relative sparsity in the shape’s interior.

Through manual segmentation of RLM images into deposit exterior, ring, and deposit interior, averages for the size and shape of the overall deposit and ring thickness were measured and are provided in the Supporting Information. An example of the segmentation and analysis of the ring and deposit interior from an RLM image is provided in the Supporting Information. The mean ring thickness was measured as the mean of the histogram of the local thickness map, where each bin count was normalized against its local thickness. Assessment of alternative measurement techniques for the ring thickness is given in the Supporting Information.

The average total deposit area (including interior) was 0.64 ± 0.06 mm^2^, and ∼16% of that was constituted by the deposited ring, whose thickness distribution had a mean of 32 ± 2 μm (averaged over observations). The high average solidity and roundness imply that an approximation of the deposit as circular may be suitable for modeling.

**Figure 3:**
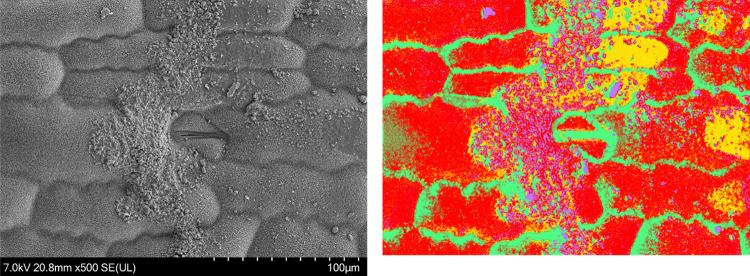
Example of semi-automated segmentation of a SEM image at 500× magnification. The 4 classes we segment into are cuticle above palisade cell (red), cuticle above cuticular peg (green), damaged cuticle (yellow), and solid particle (purple).

The greater resolution of cryo-SEM allowed the characterization of the solid fungicide particles themselves. Semi-automated segmentation using the Trainable Weka Segmentation plug-in^34^ for Fiji^32,33^ classified images into 4 classes: cuticle above palisade cell, cuticle above cuticular peg, damaged cuticle, and solid particle, an example of which is shown in Figure 3.

A particle density of 1.2 ± 0.03 μg cm^–2^ in the interior of the deposit was estimated from the mean particle volume of 2.3 μm^3^ (calculated by approximating the particles as ellipsoids on a flat surface using the projected area and minor axis length), the density of solid fungicide (1.5 g/cm^3^ by calculation and as corroborated by literature prediction^42^), the number of particles observed in a representative area of the deposit interior, and the average area of the deposit interior (0.54 ± 0.06 mm^2^). The total mass of particles in the deposit interior was thereby inferred, from which a particle density of 58.4 ± 7.1 μg cm^–2^ within the ring was calculated using the total mass expected on the surface derived from the uptake kinetics study (presented below). An interparticle separation of 17.2 ± 0.5 μm in the interior region was measured. We calculate that ∼90% of the deposited material by mass is in the ring after drying. Direct inference of the particle volumes and thus density in the coffee-ring region from the images was not performed due to the stacking of particles complicating the segmentation, alongside the inaccuracy of approximating these particles as on a flat surface when calculating their individual volumes.

The recorded images provide evidence for a significant coffee-ring effect^43−46^ during droplet drying. This is commonly seen for colloidal suspensions during drying, whereby fluid evaporation induces flow toward the droplet edge, preferentially depositing suspended particles at the triple-interface point. Surface heterogeneity also results in pinning of the droplet radius, leading to predominant Constant Contact Radius (CCR) drying,^45,46^ which was seen during our observations of the droplet evaporation. The dimensions of the coffee ring thus evidence the size and shape of the droplet–cuticle contact during CCR drying. We thus infer significant convection within the droplet during drying and a predominantly constant contact area. The effect of the convection is expected to cause homogenization of the concentration of dissolved fungicide within the droplets, permitting helpful simplification in the modeling of the kinetics of uptake, to which we next turn.

### Uptake Assay Results

Measurement of the transfer of fungicide from the surface into the leaf via uptake was undertaken for developing and fitting a kinetic model for the uptake. By measuring the mass of material within different leaf “compartments” over time after the application of fungicide as a droplet array, as described in the Methods section, experimental data are obtained to inform the model design and fit simulation.

**Figure 4:**
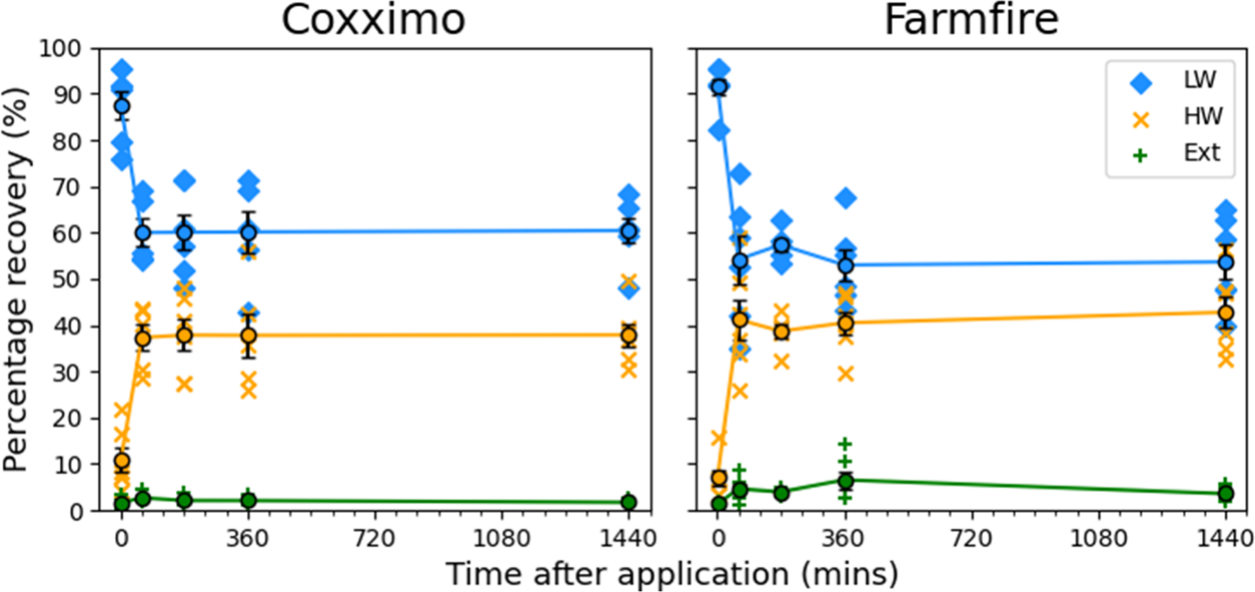
Percentage of total mass recovered from the leaf wash (LW), from the heptane wash (HW), and extracted from the remaining leaf (Ext). Averages at each time point are plotted as colored circles with black borders, and the interpolated line between them is included. Standard error bars are included in black. The result for Farmfire at the 360 min time point appears anomalous due to demonstrating a statistically significant difference in mean (*p* < 0.05) while being inconsistent with the lower Ext mass percentage values recorded at the 1440 min time point.

The uptake behavior of the fungicide within the first 24 h after droplet application was first assessed. The study consisted of three sampling processes as described in the Methods section: a leaf surface wash (LW) involving washing the surface of the treated leaf with 80:20 water/acetonitrile, a leaf surface wash with heptane (HW), and an extraction from the remaining leaf (Ext) after maceration and centrifugation with 20:80 water/acetonitrile. Two varieties of maize were studied—Farmfire and Coxximo—in order to assess the generality of the uptake results. The results are presented in Figure 4 as percentages of the total mass recovered for a given plant for each sampling process, with raw data provided in the Supporting Information. The percentage recovery from sample *i* is defined as *m*_*i*_/*m*_Tot_, where *m*_*i*_ is the mass of material recovered from sample *i* and *m*_Tot_ is the total mass recovered from all samples of the plant.

There was a short initial period of uptake within the first hour, after which the uptake plateaued to a constant value within experimental error. It has previously been observed that uptake does continue beyond 24 h [unpublished data, Syngenta] but with a very much reduced uptake rate. This corroborates our observations.

From these data, we conclude that differentiation between material bound in the cuticle and material bound in the sub-cuticle using a heptane wash was not valuable for further study. The percentage recovery of fungicide from the Ext fraction was negligible (for reliable data) relative to the other fractions and did not increase noticeably with time, implying that negligible penetration into the sub-cuticle occurs on this timescale. Additionally, there is evidence that a heptane wash may not fully remove the cuticular bound material reliably. Further discussion is presented in the Supporting Information.

We next sought to test whether the uptake results for the Coxximo and Farmfire maize varieties were statistically significantly different. *P*-tests were performed between the results of the two varieties at each time point. No significant differences (*p* ≫ 0.05 at every time point) were observed. We infer that these two maize varieties do not have a statistically significant effect on the fungicide uptake for studies of this scale and that uptake results can be generalized across maize varieties to this level of experimental error.

We then collected low-variance, short-time data in order to accurately characterize the fungicide’s uptake behavior within 1 h of application and thus develop a testable uptake model. Short-time data have larger variance due to greater relative uncertainty in the time domain for a given experimental procedure. As such the methodology for the following studies was adjusted as follows: removal of the heptane wash for greater throughput and improved time precision, total randomization of the batch order for greater data robustness, and recording of timings for droplet application and leaf sampling individually to give greater accuracy and a measure of uncertainty in time.

**Figure 5:**
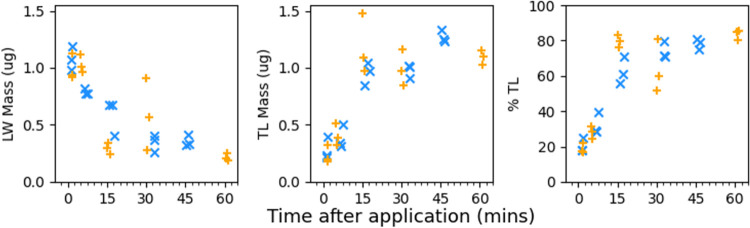
Data derived from a study of the uptake of SC fungicide on Coxximo maize. The first plot presents masses recovered from the leaf wash (LW), the second plot presents masses recovered from the remaining leaf (TL), and the third plot presents the percentage of total mass recovered as TL (%TL). The 2 batches for each time point are differentiated by marker color and shape (× and +). There is no correlation between marker selection and batch order.

We quantified the masses of material removed by a wash of the leaf surface with 80:20 water/acetonitrile (LW), the masses of material extracted from the remaining leaf after washing (TL), and the percentage of the total mass recovered from the TL fraction (%TL). The method for the application of the fungicidal droplets was unchanged. Results for Coxximo are presented in Figure 5.

The uptake followed a rapid initial uptake within the first 15 min, a similar timescale to dry-down for the formulation (10.9 ± 0.05 min).

We conclude that there are two uptake regimes: an initially rapid uptake rate within the first 15 min before a transition to a much slower uptake rate. The uptake appears to be confined within the cuticular layer with minimal penetration to the sub-cuticle during this time frame. The timescale of the initial uptake regime effectively coincides with the dry-down time for this formulation. This insight, alongside the quantitative data and surface characterizations, will inform the development of a simple model.

## Theory

**Figure 6:**
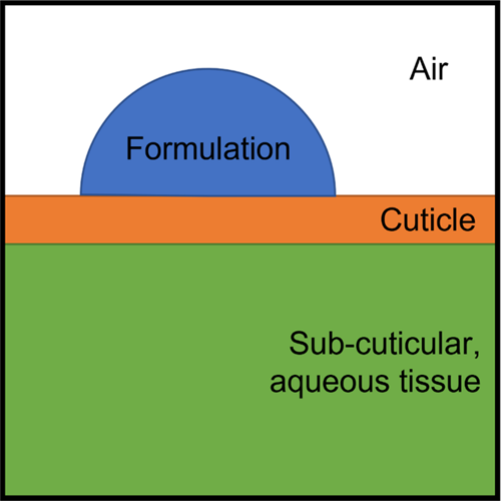
Schematic illustration of the model formulation-plant system as homogeneous compartments. Not to scale.

In this section, we develop a “simplest viable model” to describe the uptake behavior of this lipophilic fungicide. We present a simple model of the leaf-cuticle system in Figure 6 in which it is assumed that the uptake occurs from the liquid droplet during the period from application to the point at which the droplet has evaporated. The droplet-leaf contact area during this time is pinned by the coffee-ring effect. The system is modeled as four homogeneous compartments: air, formulation droplet, cuticle, and subcuticular tissue. The cuticle and sub-cuticle are modeled as uniformly flat. The contact area between the formulation droplet and cuticle is modeled as constant. The uptake through the cuticle is modeled as diffusional. The concentration within the droplet is modeled as saturated and homogeneous due to convective currents induced by evaporation and the overlapping of diffusion zones even without this convective consideration, further discussed below.

Our approach follows that commonly utilized in the literature, including by Mercer,^20^ Trapp,^19^ and our prior related work.^30^ However, we approach this as a two-dimensional system as opposed to one and consider the uptake of a highly lipophilic compound confined to the cuticle space rather than hydrophilic species that readily permeate into the sub-cuticle at the internal boundary.

Importantly, during the experiment, we ensured each droplet was sufficiently separated (≥3 mm) from neighboring droplets such that each droplet could be treated as independent. Quantitative justification for this is given in the Supporting Information. Thus, total uptake during our study can be considered to occur as a sum of independent uptakes from individual droplets. The total uptake behavior reflects the average individual droplet behavior summed over 20 droplets.

Crucially, a relatively dense and thick coffee-ring deposit shape was observed for each droplet after drying. From this, we can infer two key insights for building the simplest viable model: strong flow effects are active within the droplet during drying,^43^ which will encourage homogenization of soluble material within the droplet, and the drying is predominantly under the constant contact radius (CCR) regime^47−49^ at a radius equivalent to that of the coffee ring.

Additionally, our uptake data illustrate that the predominant uptake occurs before evaporation is complete (∼11 min for the formulation observed) and thus while the particulate material is surrounded by an aqueous solution. Previous modeling has demonstrated that foliar uptake from a particulate system within an aqueous external environment may involve the competition of two uptake pathways:^30^ a direct pathway directly through the particle–cuticle contact and an indirect pathway via dissolution into and diffusion through the aqueous environment surrounding the particles. It was predicted that the indirect uptake pathway dominates the direct uptake pathway for typical pesticides according to their physicochemical properties. A simple expression for determining whether the two uptake pathways coexist before droplet dry-down and depletion of particles was presented:  is required for simultaneous uptake. *D*_cut_ and *D*_aq_ are the diffusion coefficients for the chemical in the cuticle and in aqueous media, respectively. *c*_sat_^cut^ and *c*_sat_^aq^ are the saturation concentrations of the chemical in the cuticle and in aqueous media, respectively. Accordingly, dominant indirect uptake is predicted for the fungicide investigated in this study (*G* < 1) and the direct particle–cuticle contact is not expected to influence the uptake.

**Figure 7:**
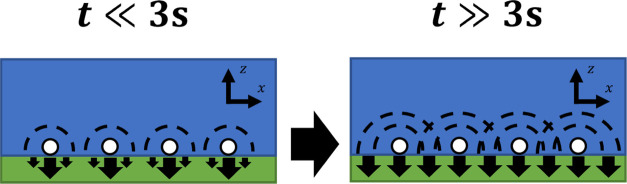
Schematic of aqueous (blue) diffusion zone (black dotted lines) overlap transition from localized to uniform aqueous concentration at the cuticle interface and its effect on the homogeneity of the transfer (black arrows) into the cuticle (green); not to scale.

The average separation of the particles in the interior region of the dried deposit was extracted from SEM images: *x*_sep_ = 17.2 μm. From this, and the approximate aqueous diffusion coefficient of *D*_aq_ = 10^–10^ m^2^ s^–1^, we can conclude that the aqueous diffusional zones between these particles significantly overlap once *t* ∼ *x*_sep_^2^/*D*_aq_ = 3 s have passed.^50−52^ Once significant aqueous diffusional zone overlap has been established, the transfer into the cuticle is effectively equivalent to uniform transfer across the entire droplet contact area since the diffusion coefficient in the cuticle is several orders of magnitude lower than in an aqueous medium.^2^ A schematic illustration is given in Figure 7.

Initially after application, the concentration of material at the cuticle interface is localized around individual particles, but after the transition to significant aqueous diffusion zone overlap at ∼3 s, the concentration at the cuticle surface, and thus the uptake rate at the surface, is uniform, assuming the uptake is partition-limited. Since this transition occurs early in the 24-h timescale we investigate, we model the second regime as being dominant throughout.

We thus model the uptake as occurring by the indirect pathway uniformly across a disk and neglect the distribution of particles on the surface beyond a consideration of their average separation. This approximation is evidently applicable before dry-down. However, as shall be demonstrated in the next section, longitudinal saturation of the cuticle occurs at the same time as dry-down and thus the uptake becomes restricted to the edge of the original interface at which radial diffusion is available. The potential localization of uptake to individual particles deposited in the ringed interior does not have an effect on the total uptake rate since the local uptake rate here is zero due to the saturation and so does not depend on the local boundary condition. As such, the approximation of a disk of uniform concentration remains viable. In cases where dry-down occurs significantly earlier than longitudinal saturation while depositing a low density of particles on the leaf surface within the coffee-ring interior (such that their cuticular diffusion zones poorly overlap) with no co-deposited medium through which indirect uptake may continue to occur, such as a non-volatile additive film, a more complex boundary condition may be required. However, this is considered to be outside the scope of this work.

**Figure 8:**
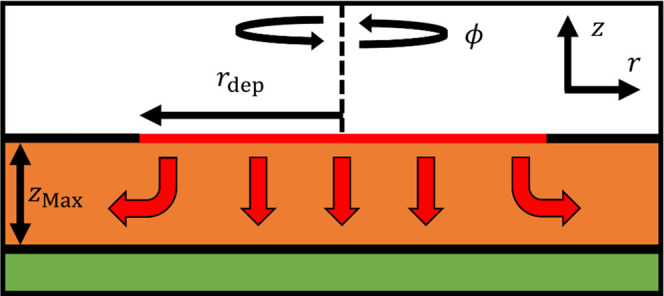
Schematic of the model cuticle system for simple simulation. A uniform, saturated disk (red) represents the cuticle-droplet contact area with a constant contact radius. Diffusion then occurs through the cuticle (orange), with the potential for a radial component to diffuse at the disk edge (represented by red arrows). Zero escape into the air (white) or subcuticular tissue (green) through zero-flux interfaces (black) corresponds to the “wax reservoir” limit. Not to scale.

We next test the simplest model that fits these observations, presented schematically in Figure 8. We approximate the droplet–cuticle interface as a uniform disk at which thermodynamic equilibrium holds (maintained by convection effects and the large ratio between the aqueous and cuticular diffusion coefficients). Within the cuticle, modeled as a thin, homogeneous layer, the material diffuses longitudinally and radially. Considering we are working with a highly lipophilic active ingredient, we apply a “wax reservoir” condition to the model whereby the material does not escape the cuticle into the air or sub-cuticle.

This problem can be represented as a partial differential equation corresponding to Fick’s 2nd Law of Diffusion.^53^ The interface between the cuticle and droplet is assumed to be saturated while each other cuticle interface does not allow transfer of active ingredient across it. Our model is conceived to be dimensionless, and thus, the predicted uptake curve is dependent on:

- *Z*_Max_ = *z*_Max_/*r*_dep_, the ratio of the cuticle thickness, *z*_Max_, and the radius of the deposit, *r*_dep_;
, the reference time which determines the dimensional timescale, where *D*_cut_ is the cuticular diffusion coefficient, *t* is the dimensional time, and *T* is the dimensionless time;
, the reference mass which converts between dimensionless and dimensional results for the penetrated mass, where *c*_sat_^cut^ is the saturation concentration of the active ingredient within the cuticle, *m* is the dimensional total mass in the cuticle, and *M* is the dimensionless total mass in the cuticle.

A larger value of *Z*_Max_ corresponds to a larger cuticle relative to the contact area with the applied formulation and fundamentally changes the uptake curve shape. A value of one for the dimensionless time or mass corresponds to a dimensional time or mass that equals the reference time or mass. The reference time is six times the time taken for a pesticidal molecule engaging in three-dimensional Brownian motion within the cuticle to have an expected square displacement equal to *r*_dep_, according to the relation ⟨*r*^2^⟩ = 6*Dt*. The reference mass is the mass of pesticide within a cube of saturated cuticle with a side length of *r*_dep_. Increasing *t*_ref_ corresponds to a slower timescale and a more slowly evolving uptake profile. Increasing *m*_ref_ corresponds to a larger mass of material taken up for an equivalent dimensionless uptake curve.

The numerical solution to this problem generates a simulation of the uptake within this model. This was performed by a backward implicit discretization scheme solved iteratively using the Biconjugate Gradient Stabilized (BICGSTAB) method and a semi-coarsening multi-grid (SMG) preconditioner. The simulation program was written in C++, utilizing the HYPRE software package (version 2.18.2)^54,55^ executed with CUDA-enabled GPU support. Construction of the discretized grid, data analysis, and plotting were performed with python, utilizing the NumPy, SciPy, and matplotlib packages.

Full descriptions of the mathematics and arrangement of the model and the techniques used to produce a numerical solution for the simulation of the active ingredient’s movement within the cuticle are given in the Supporting Information section.

## Fitting and Simulation

### Dimensionless Simulation Results

**Figure 9:**
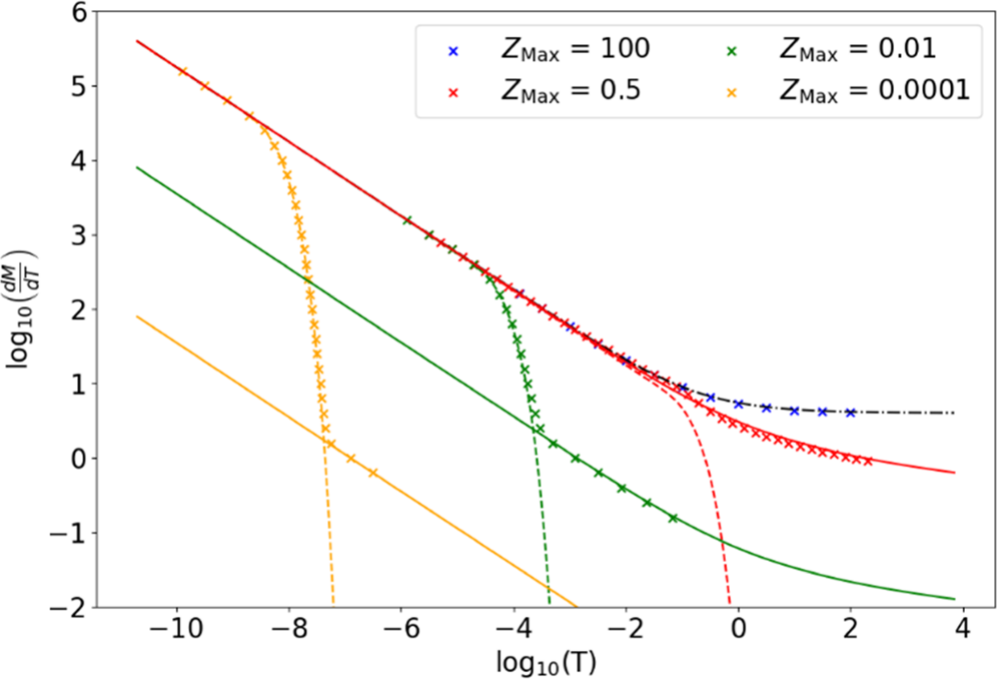
Gradient of the dimensionless total flux with dimensionless time as a function of the cuticle thickness *Z*_Max_ from simulated results. Plots of the Shoup–Szabo (dot-dashed black) and scaled Aoki equations (solid lines of color corresponding to *Z*_Max_) are included. The analytical result for a 1D system with no radial diffusion is also included as dashed lines of color corresponding to *Z*_Max_.

Dimensionless results were generated from the above model showing the dependence of the uptake rate on *Z*_Max_ for a wide range of possible values, where *Z*_Max_ is the thickness of the cuticle relative to the interfacial area. These results are presented in Figure 9 where the uptake rate is presented as , where *M* is the dimensionless mass within the cuticle and *T* is the dimensionless time, against log_10_(*T*) for a series of *Z*_Max_ values between 10^–4^ and 100. This is done to show uptake rate curves that display log–log linear initial decay before a transition to a new regime and ones that show a positive deviation from the log–log linear decay before that transition.

The dimensionless results demonstrate two limiting uptake rate regimes with flux differences of several orders of magnitude. The first is the Cottrell equation^56^
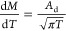
1where *A*_d_ is the dimensionless area of the disk contact (= π in the dimensionless system). This equation analytically describes linear diffusional uptake and therefore the uptake rate predicted by a one-dimensional model. For longer times, edge diffusion at the disk circumference develops, as described by the empirical Shoup–Szabo equation^57^
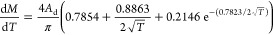
2which describes the uptake rate by diffusion from a disk into an infinite volume. This initially follows the Cottrell equation but increases at an intermediate time due to the increasing relative radial contribution to diffusion. This leads to an eventual steady-state uptake rate. This is presented in Figure 9 as a black dot-dashed line and describes the early uptake behavior.

Once *T* ∼ 0.5 × *Z*_Max_^2^, the uptake curve predicted by the model decreases. This is caused by the longitudinal saturation of the cuticle, whereby the volume between the disk contact and the internal cuticle boundary becomes saturated. This problem has been solved analytically in one-dimension by Crank^58^ to give the following co-convergent infinite series, which have been included in Figure 9 as colored dashed lines
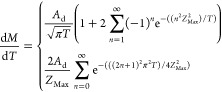
3The first infinite series converges most rapidly for *T* < *Z*_Max_^2^, and the second converges most rapidly for *T* > Z_Max_^2^. However, unlike these equations, which neglect radial diffusion, we see that the simulated uptake rates do not decay to zero but instead tend to a different limit. All uptake rate curves for this model at long dimensionless times tend to scaled versions of the Aoki equation,^59^ included in Figure 9 as colored solid lines
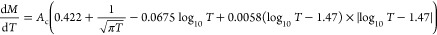
4where *A*_c_ = 2π*Z*_Max_ is the dimensionless surface area of a cylinder extruded from the disk contact to the internal cuticle boundary. The Aoki equation describes the uptake rate curve for diffusion from a uniform, cylindrical surface. Once longitudinal saturation occurs, the only means by which uptake can continue is by radial diffusion, and thus, the limiting step to uptake is diffusion from a saturated cylinder of material beneath the contact area, with a radius of *r*_dep_ and a length of *z*_Max_. This is the uptake rate’s limiting regime at long times.

The larger the value of *Z*_Max_, the later the transition from the short- to the long-time limits, since the transition begins once *T* ∼ 0.5 × *Z*_Max_^2^. The short-time limit is a uniform disk in infinite volume (eq 2), and the long-time limit is a uniform cylindrical surface of length *z*_Max_ and radius *r*_dep_ (eq 4). Small *Z*_Max_ values (≪0.5) have a transition described by eq 3 until  approaches eq 4. *Z*_Max_ = 0.5 corresponds to the cases where eqs 1 and 4 overlap at short times. For increasing *Z*_Max_, the transition to the long-time limit is more prolonged as the cylinder beneath the droplet contact/disk requires more time to saturate.

In this analysis, we present this model generally for a full range of possible *Z*_Max_ values and present equations that describe the kinetic limits: these can be used to estimate these curves of uptake rate with time without requiring further simulation. We expect that the range of *Z*_Max_ values presented here is agronomically relevant considering the wide range of cuticle thicknesses between tens of nanometers to tens of microns and the typical droplet volumes on the scale of tens to hundreds of nanolitres with highly variable wetting properties.

For fitting to our experimental data, we adopted the measured average *r*_dep_ = 450 ± 30 μm, approximating the droplet contact as a disk with the same deposit area as characterized from the images presented in the Experimental Results section. The *z*_Max_ for cuticles of maize is reported^60^ as 50 nm. From this, we infer a value of *Z*_Max_ = 1.1 × 10^–4^. The transition from a high uptake regime to a slow uptake regime observed in the experimental data can thus be explained as longitudinal saturation in the cuticle, according to this model. By fitting the dimensionless uptake rate curve simulated for this *Z*_Max_ to our experimental data, we can derive values for expected *t*_ref_ and *m*_ref_ values and thus predict *D*_cut_ and [A]_sat_^cut^ values, which can be compared with expected values to assess the feasibility of this model. This is done in the following section.

### Fitting Model Results to Uptake Assay Data

**Figure 10:**
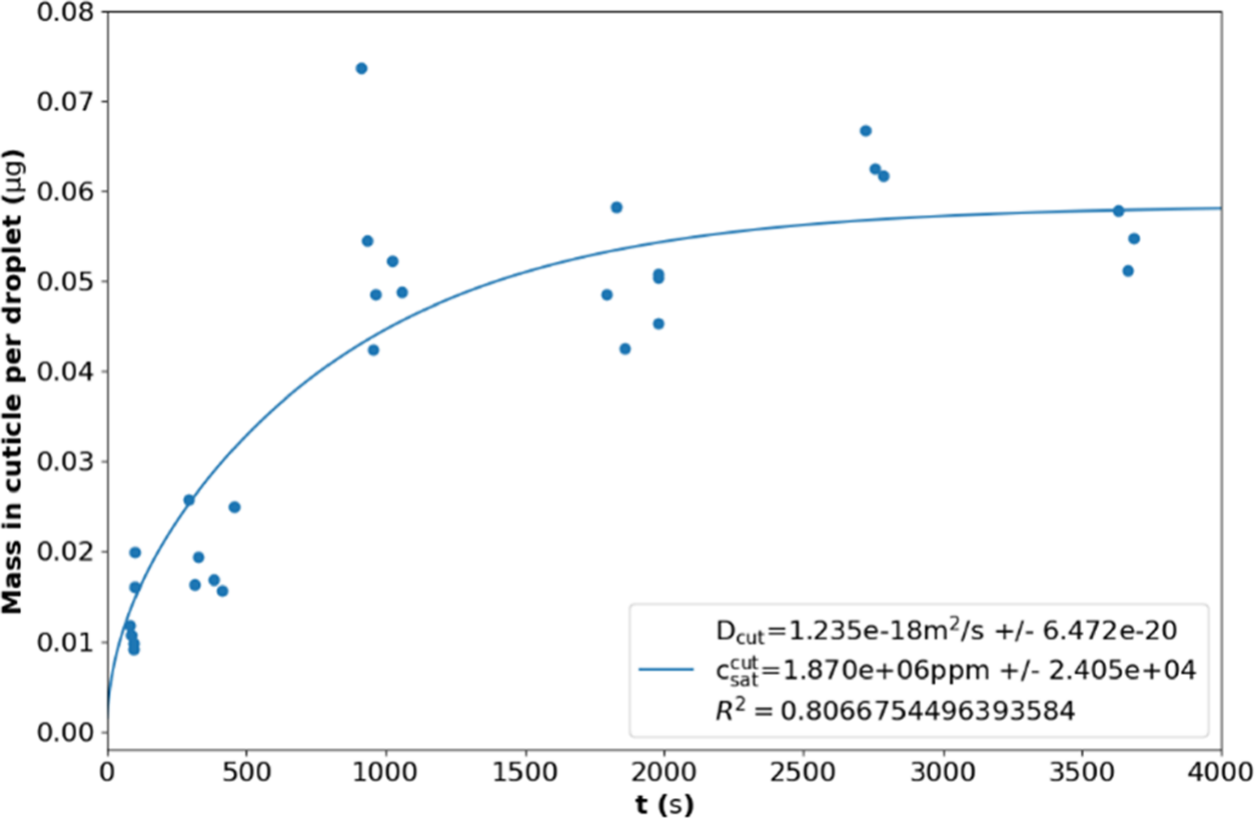
Fitting the simulated uptake rate curve for *Z*_Max_ = 1.1 × 10^–4^ to experimental data from the uptake assay study. The *D*_cut_ and *c*_sat_^cut^ values inferred from the fitted *K*_t_ and *K*_m_ values, their inferred errors and the *R*^2^ value of the fitting are presented in the Figure Legend.

For a fixed value of *r*_dep_, *t*_ref_, and *m*_ref_ are orthogonal and independently scale the time and mass dimensions, respectively. We performed a simple fitting of the simulated uptake rate curve for *Z*_Max_ = 1.1 × 10^–4^ using the Trust Region Reflective algorithm to minimize the least-squares error against the experimental data for the uptake study using Python and the scipy package. This fitting is presented in Figure 10. We have included the inferred *D*_cut_ and *c*_sat_^cut^ values with standard errors and the *R*^2^ value in the Figure legend.

A large value for *R*^2^ suggests that this model provides a good fit for this data. The model implies *D*_cut_ = (1.24 ± 0.06) × 10^–18^ m^2^ s^–1^ and *c*_sat_^cut^ = (1.87 ± 0.02) × 10^6^ ppm from fitting to our experimental data. Our value for *D*_cut_ aligns well with literature measurements for diffusion through reconstituted cuticles,^2^ suggesting that the timescale of uptake is well-described within this model and that longitudinal saturation of the cuticle is a viable explanation for the change in uptake rate.

We now consider the inferred *c*_cut_^sat^ value. A simple and common method to estimate the saturation concentration in the cuticle is to multiply the experimentally determined aqueous solubility by the partition coefficient from water into the cuticle: *K*_cw_. *K*_cw_ is often approximated using the partition coefficient of the chemical from water into a system that models the cuticular environment.^2,61−64^ If we use ethyl acetate as the model system, we expect *c*_sat_^cut^ = 1.3 × 10^5^ ppm, which is almost within one order of magnitude of the value inferred from the model. This is despite the limitations of using ethyl acetate as a solubility model, which lacks the chain lengths and secondary alcohol groups of the esters found in maize cuticle wax and cutin^60^ and thus is an encouraging comparison for the feasibility of this model.

We justify ethyl acetate as a cuticular model in this case due to the dominant presence of esters in the cuticle wax and cutin of mature maize leaves.^60^ While *K*_ow_, the octanol–water partition coefficient, is a standard model parameter to use for estimating *K*_cw_ and thus the cuticular solubility, it would predict a *c*_sat_^cut^ value of 9.5 × 10^3^ ppm, which is much lower than predicted by the model and impossible to justify considering the cuticular volume accessible per droplet in the measured time frame and the mass measured as taken up within it. This implies that comparison to octanol would be inappropriate for the case of this lipophilic fungicide and maize leaf and that ethyl acetate acts as a more suitable solubility model.

An aspect that is currently neglected by the model is the cuticular peg between the epidermal cells, an area which has a greater cuticular depth than the main cuticle proper. This provides an additional volume into which the lipophilic fungicidal material may be taken up and may explain the larger-than-expected mass of fungicide taken up, leading to an erroneously large, inferred value for *c*_sat_^cut^. The area of the cuticle above which there is cuticular peg can be observed from SEM images and has been characterized. The cuticular pegs have an average width at the outer surface of 4 ± 0.1 μm. If we assume that the cuticular peg volume below the deposit is also saturated after longitudinal saturation, we predict an average cross-sectional area of 19 μm^2^, suggesting that the depth of the cuticular peg would need to be on the order of 5 μm, which is viable for the maize cuticle.

As such, the fitted behavior of this model is entirely consistent with the partitioning of fungicide into ethyl acetate and the presence of cuticular pegs. We propose this model as the simplest viable solution for predicting the foliar uptake of lipophilic species. The model can be generalized for any *r*_dep_, *z*_Max_, *D*_cut_, and *c*_sat_^cut^ or used to infer these values from measured uptake. We present two limiting regimes for the “wax reservoir” mode of uptake and inferred equations for the calculation of their uptake rates (eq 2 and eq 4) without the need for numerical simulation. For the case of *z*_Max_ ≪ *r*_dep_, eq 3 is also provided to describe the transition between the two limiting regimes.

## Supporting Information Available

Quantitative results for the characterization of the SEM images; discussion on the method for measuring the ring thickness; the raw data and additional statistical analysis of the preliminary uptake study; justification for the droplet separation chosen; and a full description of the model (PDF)
