# Supplementary material for: Kinetics of Lipophilic Pesticide Uptake by Living Maize
Source: ACS Agric Sci Technol. 2023 Apr 18;3(5):445–54. doi: 10.1021/acsagscitech.3c00042 (PMC10189725; doi:10.1021/acsagscitech.3c00042)
Supplement: Supplementary file 1 — as3c00042_si_001.pdf [file as3c00042_si_001.pdf]

Kinetics of lipophilic pesticide uptake by living maize: Supplementary Information

*Joseph R Elliott[1], Joseph Cortvriend[2], Giovambattista Depietra[2], Colin Brennan[2], Richard G Compton[1]**

[1]Department of Chemistry, Physical and Theoretical Chemistry Laboratory, University of Oxford, South Parks Road, Oxford, OX1 3QZ, Great Britain

[2]Jealott's Hill International Research Centre, Syngenta Ltd, Bracknell, Berkshire, RG42 6EY, Great Britain

Quantitative characterisation of SEM images

Reflected Light Microscopy (RLM) and cryo-SEM (Scanning Electron Microscopy) images were taken of deposits of 0.2μL droplets of 375ppm fungicide after 1 hour of drying. RLM images were manually segmented into the coffee ring, deposit interior and deposit exterior. The cryo-SEM images were semi-automatically segmented into cuticle above palisade cell, cuticle above cuticular peg, damaged cuticle, and fungicide particle using the *Trainable Weka Segmentation*[1] plug-in for the *Fiji* package[2] *ImageJ2*[3]. The methods used to achieve this are described in the main text. The workflow used to manually segment the RLM images is illustrated in Supplementary Figure 1.

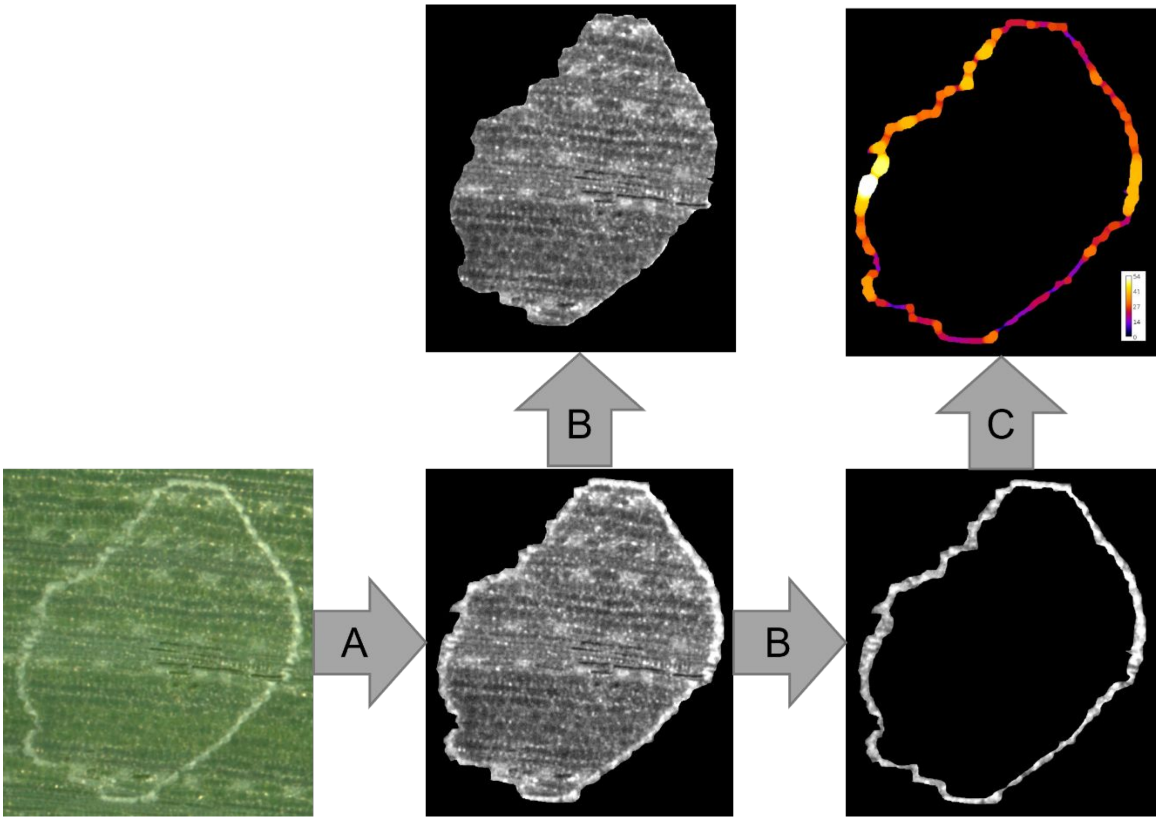

Supplementary Figure 1: Illustration of the analysis of RLM images at 40× magnification for extraction of data related to size and shape of deposits. Step A applies blue filter and manual segmentation of deposits from leaf background. Step B involves the manual segmentation of the deposit interior from the ring. Step C involves the conversion of the ring segmentation to a mask and the calculation of the local thickness map from which a histogram is derived for the calculation of the ring thickness distribution around the deposit perimeter. A colour scale is included.

These segmented images were then quantitatively analysed. Average values and standard errors for properties of the total deposit were extracted from RLM images and presented in Table 1. Average values and standard errors for properties of the leaf surface and deposited particles were extracted from cryo-SEM images and presented in Table 2 and Table 3, respectively.

Table 1: Properties extracted from optical microscopy images for 0.2µL 375ppm droplet deposits on Coxximo via manual segmentation. Results are given as averages with standard errors.

Total deposit area (mm^2)	Interior deposit area (mm^2)	Deposit aspect ratio	Deposit Circularity	Deposit Roundness	Deposit Solidity	Average coffee ring thickness $(\mu\mathrm{m})$
0.64 ± 0.06	0.54 ± 0.06	1.40 ± 0.05	0.55 ± 0.06	0.72 ± 0.02	0.91 ± 0.02	32 ± 2

Table 2: Leaf surface properties extracted from scanning electron microscopy images for 0.2µL 375ppm droplet deposit on Coxximo via semi-automated ML segmentation. Results are given as averages with standard errors.

Cuticular peg width at outer surface (µm)	Pavement cell length (µm)	Pavement cell width (µm)	Stomatal complex length (µm)	Stomatal complex width (µm)
4.0 ± 2.7	124 ± 6	42 ± 3	30.5 ± 0.97	42 ± 1.4

Table 3: Deposited particle properties extracted from scanning electron microscopy images for 0.2µL 375ppm droplet deposit on Coxximo via semi-automated ML segmentation. Results are given as averages with standard errors.

Projected area per particle in interior region $(\mu\mathrm{m}^2)$	Particle minor axis length in interior region (µm)	Particle volume in interior region $(\mu\mathrm{m}^3)$	Particle aspect ratio
1.93 ± 0.03	1.44 ± 0.01	2.30 ± 0.04	1.8 ± 0.2
Inferred particle density in interior region $(\mu\mathrm{g\ cm}^{-2})$	Inferred particle density in coffee ring region $(\mu\mathrm{g\ cm}^{-2})$	Area per particle in the interior region $(\mu\mathrm{m}^2)$	Interparticle separation in the interior region (µm)
1.2 ± 0.03	58.4 ± 7.1	295 ± 8	17.2 ± 0.5

Table 3 contains values that were calculated rather than directly measured from the images. The volumes of particles in the interior region were calculated by approximating the particles as ellipsoids on a flat surface and using the measured values for the particles' projected area, A_{proj}, and minor axis length, x_{minor}, in the following equation:

$$V(\mu\mathrm{m}^3) = \frac{4A_{\mathrm{proj}}}{3} \times \frac{x_{\mathrm{minor}}}{2}$$

The inferred particle density in the interior region, $\Gamma_{\mathrm{particle}}^{\mathrm{interior}}$, was calculated from the calculated particle volume, the density of solid fungicide ($\rho = 1.5\ \mathrm{g/cm^3}$), and the number of particles observed in a representative area, N_{rep}, of size, A_{rep}:

$$\Gamma_{\mathrm{particle}}^{\mathrm{interior}}(\mu\mathrm{g\ cm}^{-2}) = \frac{\rho \times N_{\mathrm{rep}} \times V}{A_{\mathrm{rep}}}$$

From this, the total mass of particles in the interior (measured area of A^{interior}) was calculated:

$$M^{\text{interior}} = \Gamma_{\text{particle}}^{\text{interior}} \times A^{\text{interior}}$$

The particle density in the coffee ring, $\Gamma_{\text{particle}}^{\text{ring}}$ was then calculated by comparison to the total mass measured on the surface from the uptake studies, M^{surface}, and the average area of the deposit constituting the coffee ring, A^{ring}:

$$\Gamma_{\text{particle}}^{\text{ring}} = \frac{M^{\text{ring}}}{A^{\text{ring}}} = \frac{M^{\text{surface}} - M^{\text{interior}}}{A^{\text{ring}}}$$

Calculation of the particle volumes and thus density in the coffee ring by measurement of the particles' projected area and minor axis lengths in that region was not performed due to the stacking of particles complicating the semi-automated segmentation of individual particles and invalidating the approximation of particles as ellipsoids on a ***flat*** surface.

An example image of the epicuticular wax crystals and solid fungicide particles is presented in Supplementary Figure 2.

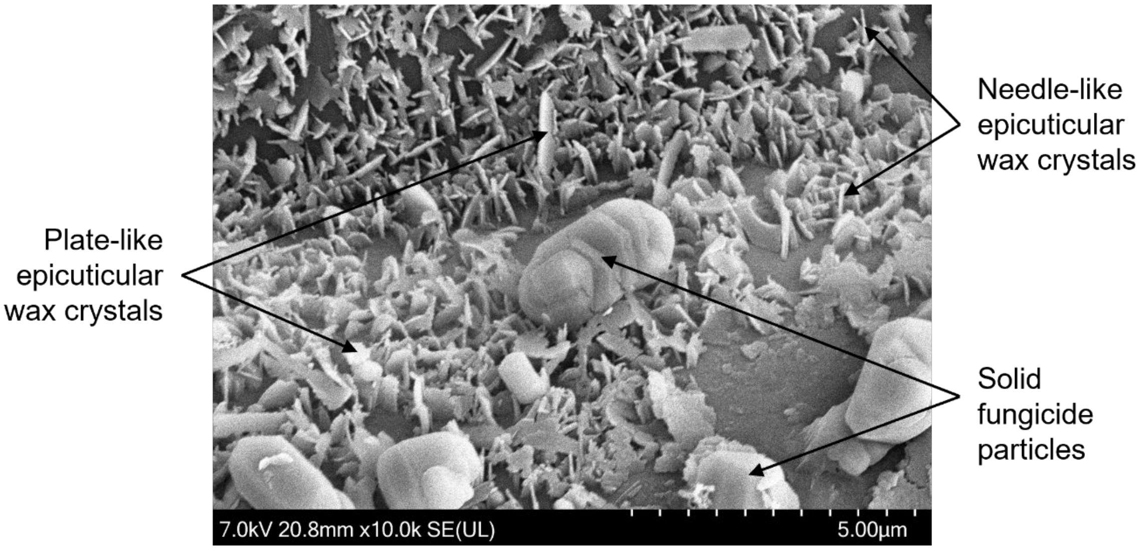

Supplementary Figure 2: Cryo-SEM image of solid fungicide particles on cuticle near a cuticular peg-induced surface trough. This illustrates the size and shape of the epicuticular crystals relative to the size and shape of the fungicide particles and their effect on the contact with the underlying smooth cuticle. Image magnification wax 10,000×. A scale bar of $5\ \mu m$ has been included at the bottom of the image.

Ring thickness measurement

We tested several methods for measuring the coffee ring thickness from RLM images of the deposits of 0.2μL droplets of 375ppm fungicide after 1 hour of drying and manual segmentation into coffee ring, deposit interior and deposit exterior. The radial thickness was measured relative to the centroid of the deposit to give a radial thickness distribution across the range $0 \le \theta < 2\pi$. The histogram of the local thickness heatmap of the mask of the coffee ring segment gives the distribution of the local thickness across the whole coffee ring area. However, it over-represents the large local thickness regions (greater local thickness results in more pixels within that area) and so the number of pixels in each bin was normalised against the local thickness value of that bin to measure the distribution of the local thickness around the coffee ring perimeter. The distance-to-nearest-edge heatmap was generated for the mask of

the coffee ring segment. A midline around the coffee ring was generated along the maximum cusp of the distance-to-nearest-edge heatmap. The distribution along this midline of local thickness and double-distance-to-nearest-edge (which equals the edge-to-edge distance at the maximum)provides alternative measures of the coffee ring thickness around the coffee ring perimeter. Example results are presented in Supplementary Figure 3.

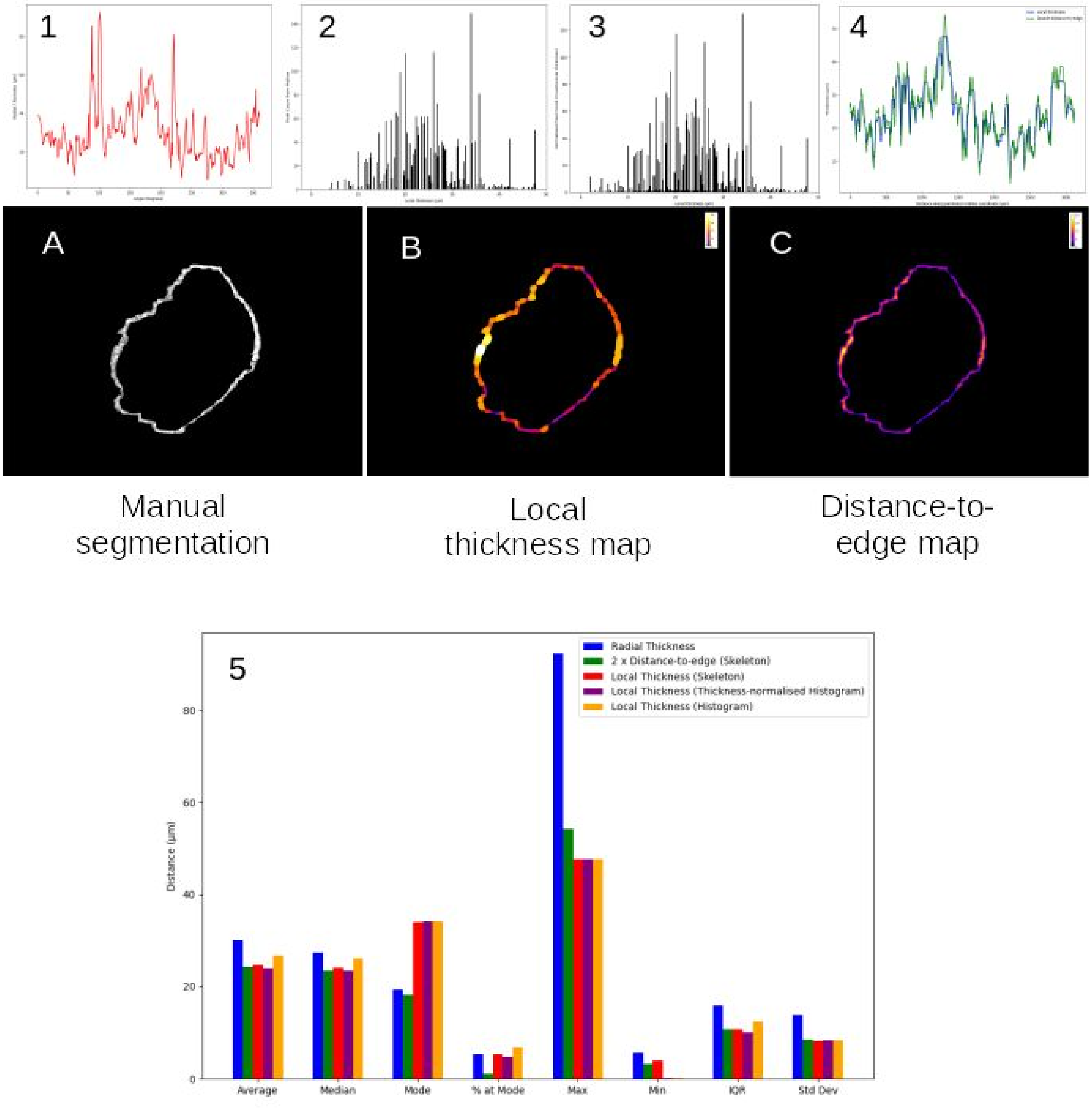

Supplementary Figure 3: Image A is the manually segmented coffee ring from a 40x magnification optical microscopy image of a $375\ ppm$ 10% IPA $0.2\mu L$ deposit on a Coxximo leaf. Image B is the local thickness heat-map of the mask of the coffee ring. Image C is the distance-to-nearest-edge heat-map of the mask of the coffee ring. Plot 1 is the radial thickness profile from the deposit's centroid. The distance-to-nearest-edge map is used to manually generate a midline. Plot 2 is the histogram of local thickness of pixels on this midline. Plot 3 is the histogram of local thickness of all pixels in the heat-map normalised against the thickness. Plot 4 is the profile of local thickness (blue) and double-distance-to-nearest-edge along the midline coordinate. Plot 5 are the statistics of the thickness distributions produced by these methods: radial thickness is blue, double-distance-to-nearest-edge along the midline is green, local thickness along midline is red, local thickness histogram normalised against the local thickness is purple, and non-normalised local thickness histogram is orange.

The radial thickness measurement overestimates the thickness due to the slight non-circularity of the deposit's coffee ring, leading to measurements of distances not perpendicular to the coffee ring edges as its thickness in certain regions; this results in larger values of the average, median, maximum, minimum, IQR and standard deviation, and is thus discarded as a viable measurement. The non-normalised local thickness histogram similarly overestimates the coffee ring thickness distribution as it considers local thickness distribution across the coffee ring area instead of its perimeter, resulting in overestimates of the average, median and IQR values. This measurement is less valuable and thus discarded. The average, median and standard deviation values of the distributions of the double-distance-to-edge along midline skeleton, local thickness along midline skeleton and normalised local thickness histogram are generally in close agreement with each other. The double-distance-to-edge distribution has a larger maximum, smaller mode and smaller % of distribution at the mode value. These 3 measurements are each viable. We chose to present the normalised local thickness histogram value as it avoids the generation of the midline, which is an additional source of error and increases the characterisation time.

Preliminary uptake study raw data and additional analysis

As described in the main text, we performed a preliminary uptake study to assess the uptake behaviour over the first 24 hours after application of an array of 20 0.2µL droplets of 375ppm fungicide on the maize leaf surface. 3 sampling processes were used: a leaf surface wash with 80:20 water:acetonitrile (LW), a heptane leaf surface wash (HW) and an extraction from the remaining leaf into 20:80 water:acetonitrile after maceration and centrifugation (Ext). Subsamples of these were scanned by LC-MS to generate a signal for the fungicide, which was integrated and compared against a calibration curve to give a concentration of the active ingredient in the sample, from which the mass contained in the sample was calculated. These calculated mass results are presented in Supplementary Figure 4.

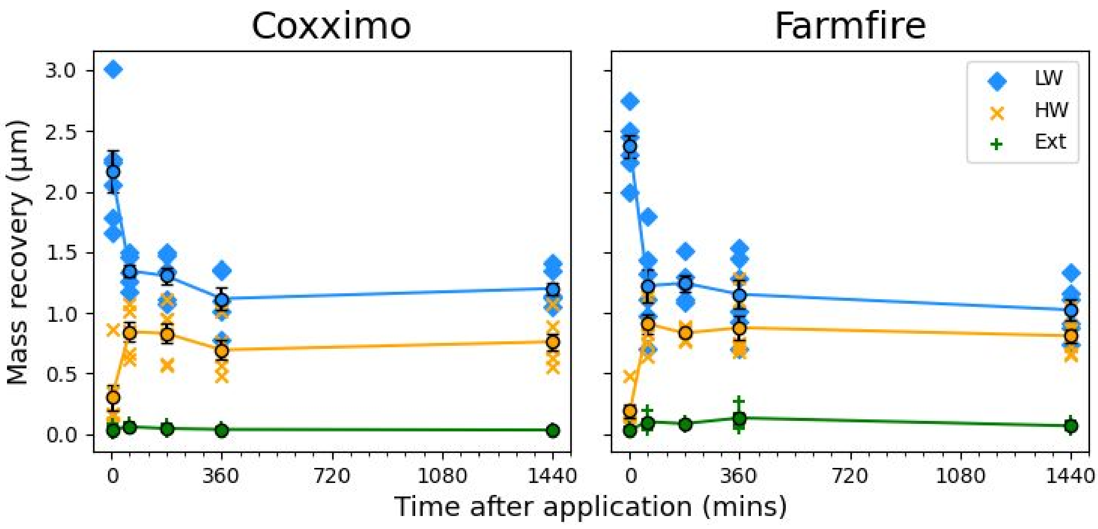

Supplementary Figure 4: Raw data for masses recovered from each sampling process (LW: blue diamond, HW: orange ×, Ext: green +) for Coxximo (left) and Farmfire (right) maize varieties. Averages at each time-point are plotted as coloured circles with black borders and the interpolated line between them is included. Standard error bars are included in black.

Supplementary Figure 4 shows a rapid initial decay in material recovered from the leaf wash (LW) matched by a rapid initial increase in material recovered from the heptane wash (HW)

and both reach a plateau. The mass recovered from the extract after the heptane wash is negligible and no statistically significant variation in time can be established.

Supplementary Figure 5 shows the results for the percentage recovery of the TL $\left(\frac{m_{\mathrm{TL}}}{m_{\mathrm{LW}}+m_{\mathrm{HW}}+m_{\mathrm{TL}}}\right)\times 100\%$ against the HW $\left(\frac{m_{\mathrm{HW}}}{m_{\mathrm{LW}}+m_{\mathrm{HW}}+m_{\mathrm{TL}}}\right)\times 100\%$ sampling methods for this preliminary study, where m_{X} is the mass recovered from sampling process X. From this we can assess visually the presence of correlation between these two values. Correlation, if any, would imply that the recovery of material using the heptane wash is incomplete and that a significant proportion of the material recovered from the extract is truly material in the cuticle compartment that had not been recovered by the heptane wash. There is not a strong correlation for the Coxximo results but there is a correlation for the Farmfire results whereby a large %HW corresponds to a large %TL. This is quantitatively evidenced by the Pearson and Spearman correlation coefficients, which measure the degree of linear and non-linear correlation respectively, presented in Table 4. We see a strong positive non-linear correlation for the Farmfire case. This provides evidence that the recovery of material from the cuticle using a heptane wash is incomplete, particularly when %HW is large, and that this leads to correlatively increased %TL values, since cuticular material has been left behind to be extracted from the remaining leaf. The %TL values for Farmfire are also significantly larger than those for Coxximo with respect to their paired %HW, with an increasing difference as %HW increases. This evidence suggests that the heptane wash is an unreliable technique for extracting material from the cuticle in the case of Farmfire, which is a driver for the exclusion of the heptane wash from the later uptake study.

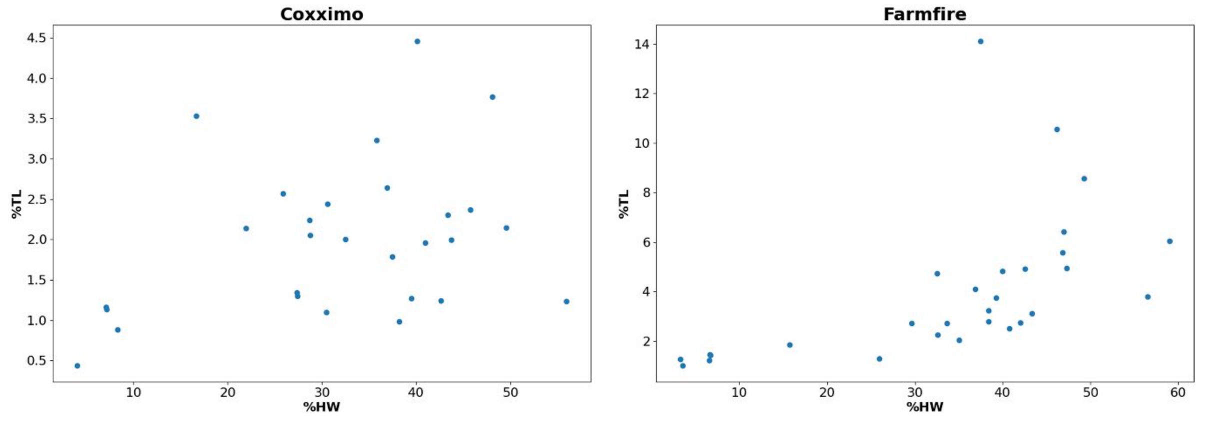

Supplementary Figure 5: Results from the Preliminary study of uptake of percentage recovery from the remaining leaf extract (%TL) versus percentage recovery from the heptane wash sampling (%HW) for Coxximo (left) and Farmfire (right).

Table 4: Correlation coefficients between %HW and %TL for Coxximo and Farmfire.

Correlation (%HW:%TL)	Pearson	Spearman
Coxximo	0.34	0.29
Farmfire	0.55	0.81

Justification for droplet separation for independent uptake per droplet

We investigated uptake from an array of droplets containing fungicide for the purpose of developing a model to describe the uptake behaviour. A droplet separation of ≥ 3mm was used to ensure that uptake from each droplet was independent. Considering that the uptake pathway in this case is astomatous diffusion through the cuticle, we can assess at what distance apart droplets must be to ensure that their diffusion zones within the cuticle do not overlap. According to the Einstein-Smoluchowski equation[4, 5], the expected square displacement of a diffusing particle after time t is: $\langle x^2 \rangle = 6Dt$ in three dimensions. As such, we can consider the root mean square displacement of the fungicide through the cuticle after 24 hours to be: $\sqrt{\langle x^2 \rangle} = 7.2 \times 10^{-7}$ m $= 0.7$μm, where we have used $D_{\text{cut}} = 10^{-18}$ m^{2}s^{-1}. If we consider the droplet radii are 450 μm, a separation of ~ 1 mm is required to avoid two droplets coalescing. If we include two diffusion zones, then one must include an additional 1.5μm separation at least to avoid the diffusion zones significantly overlapping. However, to ensure no interference, a minimum centre-to-centre separation of 3mm was used. This ensures that over the course of 24 hours, a diffusing particle would not expect to interfere with the uptake at an adjacent droplet if its diffusion coefficient is 2×10^{-12} m^{2}s^{-1} or less. Given the measured and expected values of the diffusion coefficient (see main text) we conclude that uptake from a single droplet is independent of the other droplets.

Additional description of the model

The model developed is based on Fick's 2nd Law of diffusion[6] in two-dimensional cylindrical coordinates, r, perpendicular distance from axis of symmetry, and z, distance from reference plane perpendicular to symmetry axis:

$$\frac{\partial c}{\partial t} = D\left(\frac{\partial^2 c}{\partial z^2} + \frac{\partial^2 c}{\partial r^2} + \frac{1 \partial c}{r \partial r}\right)$$

where c is the concentration and D is the diffusion coefficient.

We implement a complete set of boundary conditions, presented in Supplementary Figure 6. We apply zero-flux boundaries at the flat cuticle's longitudinal interfaces, which we justify in the main text as applying the "wax reservoir" limit such that no material is lost from the cuticle into the air or sub-cuticle. We define a r_{Max} value as the simulation's radial limit at which we also apply a zero-flux boundary. r_{Max} is defined in relation to t_{Max}, the largest value in time to which we simulate, such that we are within the infinite limit and r_{Max} does not affect the solution: $r_{\text{Max}} = 6\sqrt{Dt_{\text{Max}}}$. This is justified in the above section by the diffusional independence of the uptake from adjacent droplets. The axis of symmetry must, by definition, also have a zero-flux boundary through it such that there is no concentration gradient across this axis. We implement a zero-concentration condition at $t = 0$, assuming no fungicide is already present within the cuticle. We implement a uniform constant-concentration boundary condition at the cuticle-droplet contact disk area, assuming that thermodynamic equilibrium is held and that the cuticle here is saturated such that $c = c_{\text{sat}}$. This set of boundary conditions is presented below:

$$\begin{cases} \frac{\partial c}{\partial z} = 0 \text{ at } (r,z) = (r > r_{\text{dep}},0) \text{ and } (r,z_{\text{Max}}) \\ \frac{\partial c}{\partial r} = 0 \text{ at } (r,z) = (0,z) \text{ and } (r_{\text{Max}},z) \\ c = 0\ \forall\{r,z\} \text{ at } t = 0 \\ c = c_{\text{sat}} \text{ at } (r,z,t) = (r \le r_{\text{dep}}, z = 0, t > 0) \end{cases}$$

where r_{dep} is the radius of the disc through which uptake occurs, corresponding to the radius of the cuticle-droplet contact area, z_{Max} is the maximum simulation longitudinal distance, which corresponds to the cuticle thickness, and c_{sat} is the saturation concentration of the fungicide in the cuticle.

In order to simplify the mathematics and generalise the results of a simulation, we convert these parameters to dimensionless values using the following conversions presented in Table 5.

Table 5: Table of dimensionless conversions for model parameters

Dimensional parameter	Dimensionless conversion
r	$R = r/r_{\text{dep}}$
z	$Z = z/r_{\text{dep}}$
r_{dep}	$R_{\text{dep}} = r_{\text{dep}}/r_{\text{dep}} = 1$
r_{Max}	$R_{\text{Max}} = r_{\text{Max}}/r_{\text{dep}}$
z_{Max}	$Z_{\text{Max}} = z_{\text{Max}}/r_{\text{dep}}$
D_{cut}	$d_{\text{cut}} = D_{\text{cut}}/D_{\text{cut}} = 1$
c	$C = c/c_{\text{sat}}^{\text{cut}}$
t	$T = D_{\text{cut}} \cdot t/r_{\text{dep}}^2 = K_{\text{t}} \cdot t$
$m = 2\pi \int_0^{r_{\text{Max}}} \int_0^{z_{\text{Max}}} c\, r\, dz\, dr$	$M = 2\pi \int_0^{R_{\text{Max}}} \int_0^{Z_{\text{Max}}} C\, R\, dZ\, dR = m/c_{\text{sat}}^{\text{cut}} r_{\text{dep}}^3 = K_{\text{m}} \cdot m$
j	$J = r_{\text{dep}} \cdot j/D_{\text{cut}} \cdot c_{\text{sat}}^{\text{cut}}$
$j_{\text{Tot}} = 2\pi \int_0^{r_{\text{dep}}} j\, r\, dr$	$J_{\text{Tot}} = 2\pi \int_0^1 J\, R\, dR = j_{\text{Tot}}/D_{\text{cut}} \cdot c_{\text{sat}}^{\text{cut}} \cdot r_{\text{dep}}$

where m is the total dimensional mass in the cuticle, j is the dimensional flux at a given position $(\text{mol m}^{-2}\,\text{s}^{-1})$ given by Fick's First Law $\left(j = -D\frac{\partial c}{\partial x}\right)$, j_{Tot} is the increase in total mass in the cuticle over time according to the integration of flux at the disk (mol s^{-1}), and $K_{\text{t}} = D_{\text{cut}}/r_{\text{dep}}^2$ and $K_{\text{m}} = \left(c_{\text{sat}}^{\text{cut}} r_{\text{dep}}^3\right)^{-1}$ are dimensionless conversion factors for mass and time, which are orthogonal for a given r_{dep}. We can now express the PDE and boundary conditions in dimensionless terms:

$$\begin{cases} \frac{\partial C}{\partial T} = \frac{\partial^2 C}{\partial Z^2} + \frac{\partial^2 C}{\partial R^2} + \frac{1 \partial C}{R \partial R} \forall (R, Z, T) \\ \frac{\partial C}{\partial Z} = 0 \text{ at } (R, Z) = (R > 1, 0) \text{ and } (R, Z_{\mathrm{Max}}) \\ \frac{\partial C}{\partial R} = 0 \text{ at } (R, Z) = (0, Z) \text{ and } (R_{\mathrm{Max}}, Z) \\ C = 0 \; \forall \{R, Z\} \text{ at } T = 0 \\ C = 1 \text{ at } (R, Z, T) = (R \leq 1, Z = 0, T > 0) \end{cases}$$

Supplementary Figure 6 presents the model proposed schematically.

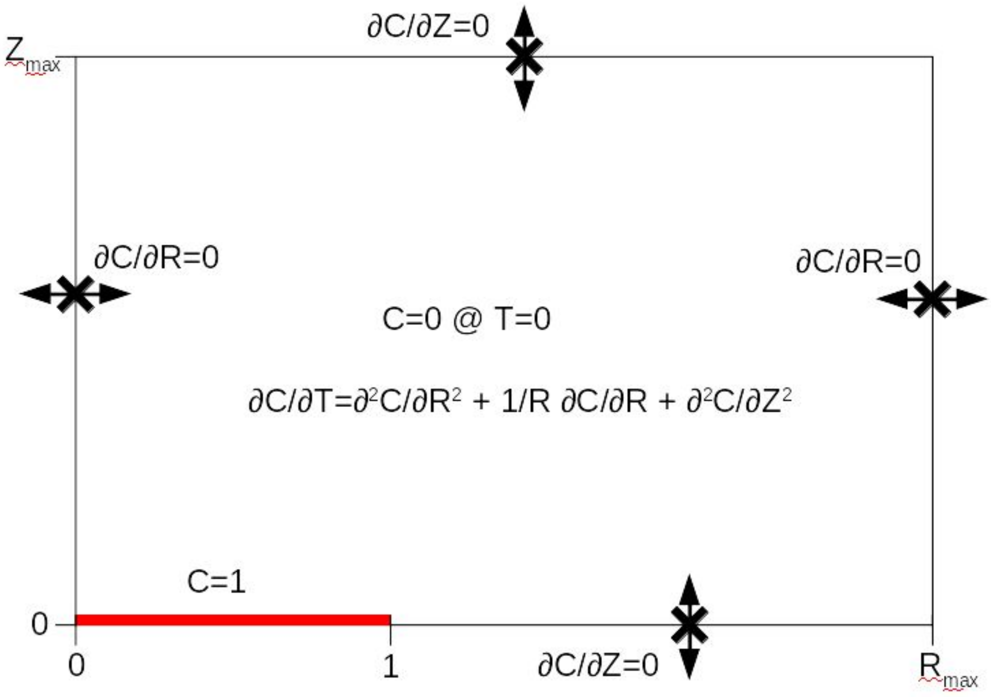

Supplementary Figure 6: Schematic of simulation space with associated boundary conditions and PDE in dimensionless form

For numerical simulation, we discretise these equations onto points on a grid and represent the PDE and boundary conditions using the finite difference method. The discretisation scheme is illustrated in Supplementary Figure 7.

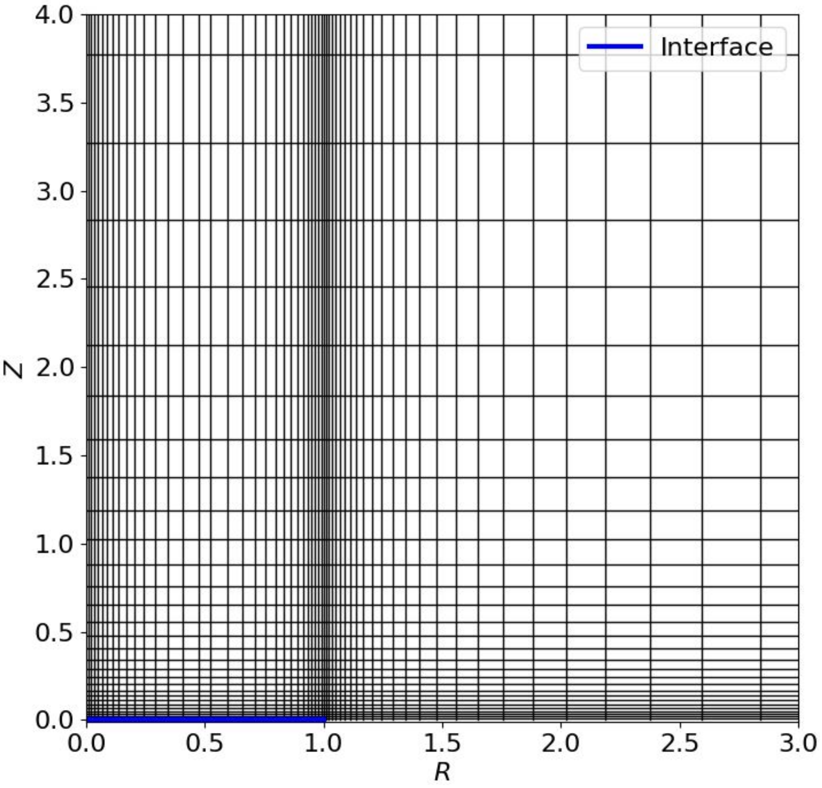

Supplementary Figure 7: Illustration of the discretisation scheme used. Parameters used are $h_R = h_Z = 0.01$ *and* $\omega_R = \omega_Z = 1.15$. *These values are chosen to be illustrative only.*

An exponentially expanding grid[7-10] was used since the concentration gradients decrease with distance from the source disc and so the density of the grid required to achieve accurate simulation also decreases with distance. The distribution of points in such a grid for a generic coordinate X is given as:

$$X_i = X_{i-1} + h_X \times (\omega_X)^{i-1}$$

where h_X is the initial step distance $(X_1 - X_0)$ and ω_X is the expansion factor. The Z and T grid-steps are monotonically expanding. The R grid-steps expand until $R = 0.5$, then contract to $R = 1$ (to give a symmetric distribution about $R = 0.5$), before expanding to $R = R_{\mathrm{Max}}$. This is done to ensure high density of points around the disc edge where concentration gradients are largest. A backward implicit discretisation[11-13] is used to resolve the steps in time.

This discretised, finite difference system is then solved fully implicitly and iteratively using the Biconjugate Gradient Stabilised method (BICGSTAB), with a relative convergence tolerance of 1×10^{-6}, with a semi-coarsening multigrid (SMG) preconditioner. The numerical simulation was performed on a *Linux(Centos)* machine with an *Intel Core i7-6800K* CPU (3.40GHz, 6 cores), 32GB of RAM, and an *Nvidia Quadro GP100*. The simulation program was written in C++, utilising the *HYPRE* software package (version 2.18.2)[14, 15] executed with *CUDA* enabled GPU support. Construction of the discretised grid, data analysis, and plotting were performed with *python*, including the *NumPy*, *SciPy* and *matplotlib* packages.

Supplementary References

1. Arganda-Carreras, I.; Kaynig, V.; Rueden, C.; Eliceiri, K. W.; Schindelin, J.; Cardona, A.; Sebastian Seung, H., Trainable Weka Segmentation: a machine learning tool for microscopy pixel classification. *Bioinformatics* **2017,** *33* (15), 2424-2426.

2. Schindelin, J.; Arganda-Carreras, I.; Frise, E.; Kaynig, V.; Longair, M.; Pietzsch, T.; Preibisch, S.; Rueden, C.; Saalfeld, S.; Schmid, B.; Tinevez, J.-Y.; White, D. J.; Hartenstein, V.; Eliceiri, K.; Tomancak, P.; Cardona, A., Fiji: an open-source platform for biological-image analysis. *Nature Methods* **2012,** *9* (7), 676-682.

3. Rueden, C. T.; Schindelin, J.; Hiner, M. C.; DeZonia, B. E.; Walter, A. E.; Arena, E. T.; Eliceiri, K. W., ImageJ2: ImageJ for the next generation of scientific image data. *BMC Bioinformatics* **2017,** *18* (1), 529.

4. Einstein, A., Über die von der molekularkinetischen Theorie der Wärme geforderte Bewegung von in ruhenden Flüssigkeiten suspendierten Teilchen. *Annalen der Physik* **1905,** *322* (8), 549-560.

5. von Smoluchowski, M., Zur kinetischen Theorie der Brownschen Molekularbewegung und der Suspensionen. *Annalen der Physik* **1906,** *326* (14), 756-780.

6. Fick, A., V. On liquid diffusion. *The London, Edinburgh, and Dublin Philosophical Magazine and Journal of Science* **1855,** *10* (63), 30-39.

7. Gavaghan, D. J., An exponentially expanding mesh ideally suited to the fast and efficient simulation of diffusion processes at microdisc electrodes. 1. Derivation of the mesh. *Journal of Electroanalytical Chemistry* **1998,** *456* (1), 1-12.

8. Rudolph, M., Digital Simulations on unequally spaced grids. Part 1. Critical remarks on using the point method by discretisation on a transformed grid. *Journal of Electroanalytical Chemistry* **2002,** *529*, 97-108.

9. Seeber, R.; Stefani, S., Explicit finite difference method in simulating electrode processes. *Analytical Chemistry* **1981,** *53* (7), 1011-1016.

10. Feldberg, S. W., Optimization of explicit finite-difference simulation of electrochemical phenomena utilizing an exponentially expanded space grid: Refinement of the Joslin-Pletcher algorithm. *Journal of Electroanalytical Chemistry and Interfacial Electrochemistry* **1981,** *127* (1), 1-10.

11. Alden, J. A.; Compton, R. G., A general method for electrochemical simulations. 1. Formulation of the strategy for two-dimensional simulations. *The Journal of Physical Chemistry B* **1997,** *101* (44), 8941-8954.

12. Compton, R. G.; Kätelhön, E.; Laborda, E.; Ward, K. R., *Understanding voltammetry: Simulation of electrode processes*. Second ed.; World Scientific Europe: 2020.

13. Laasonen, P., Über eine Methode zur Lösung der Wärmeleitungs-gleichung. *Acta Mathematica* **1949,** *81*, 309-317.

14. Falgout, R. D.; Yang, U. M. In *hypre: A Library of High Performance Preconditioners*, Berlin, Heidelberg, Springer Berlin Heidelberg: Berlin, Heidelberg, 2002; pp 632-641.

15. Falgout, R. D.; Jones, J. E.; Yang, U. M. In *The Design and Implementation of hypre, a Library of Parallel High Performance Preconditioners*, Berlin, Heidelberg, Springer Berlin Heidelberg: Berlin, Heidelberg, 2006; pp 267-294.
